# Association of sexual pain and psychological factors among gynecologic and breast cancer patients: application of components of the fear-avoidance model of chronic pain

**DOI:** 10.1007/s10865-025-00560-3

**Published:** 2025-03-13

**Authors:** Lora L. Black, Katherine Conroy, Maryam Lustberg, Ritu Salani, Barbara L. Andersen, Kristen M. Carpenter

**Affiliations:** 1 Department of Psychiatry & Behavioral Sciences, Wexner Medical Center, The Ohio State University, Columbus, OH USA; 2 Department of Psychology, University of Washington, Seattle, Washington USA; 3 Department of Internal Medicine (Medical Oncology), Yale School of Medicine, New Haven,, CT USA; 4 University of California Los Angeles Obstetrics and Gynecology, Los Angeles, CA USA; 5 Department of Psychology, The Ohio State University, Columbus, OH USA

**Keywords:** Gynecological cancer, Breast cancer, Sexual health, Pain

## Abstract

A significant number of gynecologic and breast cancer survivors report chronic issues with pain during sexual activity. The fear-avoidance (FA) model of chronic pain provides a potential framework for addressing chronic sexual pain. The purpose of this study is to investigate the relationships among components of the FA model (acute pain, anxiety, avoidance, and distress) among gynecologic and breast cancer survivors to help identify those who may be at risk for chronic sexual pain. Gynecologic and breast cancer patients (*n* = 97) completed baseline questionnaires as part of a psychosexual intervention. Linear regression model was used to test components of the FA model. Overall, 17–34% of female cancer survivors experienced pain related to sexual activity in the month prior to enrolling in a psychosexual intervention trial. Further, 51% of participants reported clinically significant levels of sexual distress. Results of a multiple linear regression show that sexual distress was significantly associated with acute sexual pain (Standardized β = 0.34, *p* <.01), anxiety (Standardized β = 0.28, *p* <.05), and avoidance of sexual activity (Standardized β = 0.28, *p* <.01) when controlling for sexual activity. Survivors of breast and gynecologic cancer entering a sexuality treatment study reported pain with sexual activity. Further, sexual distress was significantly associated with acute sexual pain, anxiety, and avoidance of sexual activity, pointing to contributions each of these FA model components have on sexual distress in this population. These findings point to the need for interventions to explicitly address anxiety and avoidance of chronic sexual pain among female cancer survivors.

Women account for approximately 50% of new cancer diagnoses; 42% of these new diagnoses are breast or gynecologic cancers (Siegel et al., 2019). Advanced treatments have improved survival (Giaquinto et al., 2022; Giordano et al., 2004), but there are significant, often chronic, treatment sequelae, with a salient one being sexual disruption and/or dysfunction (Bober & Varela, 2012; Mercadante et al., 2010; Sadovsky et al., 2010; Schover, 2005). Up to 80% of gynecologic and 30–100% of breast cancer survivors report at least one sexual side effect of treatment (Bober & Varela, 2012), with complaints of pain related to sexual activity among the most commonly reported problems (Falk & Dizon, 2013; Krychman & Millheiser, 2013). Indeed, 35–38% of breast (Bober & Varela, 2012) and 29–40% of gynecologic cancer (Bergmark et al., 1999; Jensen et al., 2003, 2004; Lindau et al., 2007; Pieterse et al., 2006) survivors report experiencing pain during sexual activity. Common treatments for gynecologic and breast cancer cause vaginal dryness and atrophy (Bergmark et al., 1999), both of which contribute to pain during sexual activity. Given the high rates of sexual morbidity, the American Society of Clinical Oncology calls for the assessment of sexual health and dysfunction among all cancer survivors at diagnosis, treatment, and follow-up (Carter et al., 2018).

Given the magnitude and chronicity of the problem, it is important to understand variables associated with onset and maintenance of sexual pain among female cancer survivors. The fear-avoidance (FA) model of chronic pain offers a potential framework for understanding and addressing this issue. Traditionally used to explain musculoskeletal pain (i.e., pain that affects bones, muscles, ligaments, tendons, or nerves), the FA model describes the role that psychological factors, notably anxiety/fear, avoidance, and pain catastrophizing interrelate and contribute to chronicity of pain (Vlaeyen & Linton, 2012). The FA model may also be applicable in women who experience pain with sexual intercourse (Thomtén & Linton, 2013). Specifically, the FA model posits that some patients interpret pain as threatening, which contributes to both increased levels of anxiety and avoidance of behaviors that they fear will lead to more pain. This strategy can be effective for reducing acute pain; however, continued avoidance of activities can lead to disuse, disability, and distress that lower the pain threshold in subsequent experiences (Vlaeyen & Linton, 2012), thus compounding the problem. Among female cancer survivors, the occurrence of pain is one of the most common and significant deterrents to sexual activity among female cancer survivors (Abbott-Anderson & Kwekkeboom, 2012). Further, preoccupation with and fear of pain in future sexual encounters is associated with decreased desire to engage in sexual activity (Reis et al., 2010; Ussher et al., 2012). As such, it is important to better understand the relationship between FA components, such as pain and avoidance, among female cancer survivors that may have sexual concerns.

There is a current lack of knowledge about the role of psychological factors associated with the experience of sexual pain in patient populations at risk for chronic sexual dysfunction, such as female cancer survivors. Use of the FA model allows for a model-driven approach to the assessment of sexual pain and conceptualization of treatment development. The aim of the present study is to investigate the relationships between some components of the FA model (pain, anxiety, avoidance, and distress) among gynecologic and breast cancer survivors as they enrolled in a psychosexual intervention trial. Specifically, we hypothesize that recent experiences of sexual pain (i.e., sexual pain that has occurred within the past month), anxiety, and avoidance of sexual activity will be positively associated with reports of sexual distress. The identification of FA components that covary with distress among female cancer survivors can help to identify those who may be at risk for the development of chronic sexual pain.

## Methods

### Procedure

**Fig. 1:**
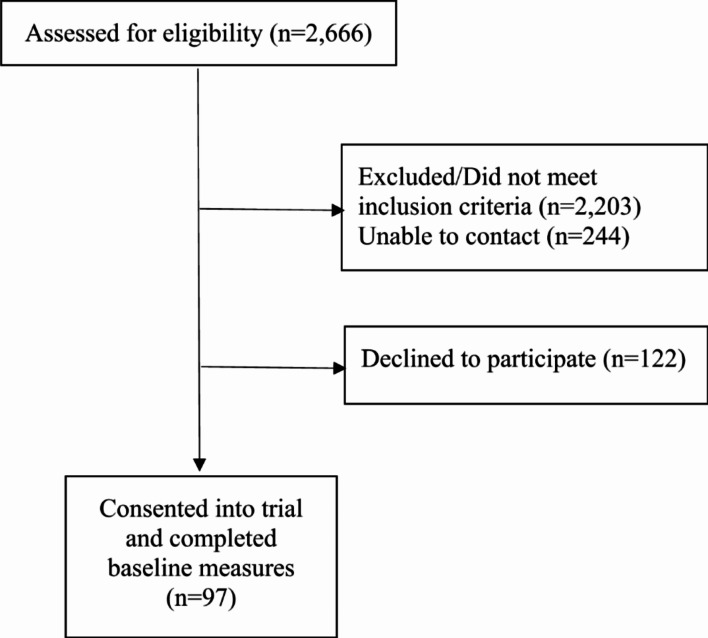
CONSORT flow diagram

This study was approved by the IRB at The Ohio State University. Participants (*n* = 97) were gynecologic or breast cancer survivors enrolling in a Phase I trial of a psychosexual intervention delivered in a group format (ClinicalTrials.gov: NCT01764802). See Table 1 for participant demographic and disease/treatment information. Potential participants were identified by clinic staff from the gynecologic and breast cancer services at a university-affiliated, National Cancer Institute Comprehensive Cancer Center, advertisements/flyers in clinics and community settings, and self-referrals. Inclusion criteria were as follows: diagnosis of stage I-III breast or gynecologic cancer, age ≥ 21 and ≤ 80, able to speak/read English, and able to provide informed consent. Exclusion criteria were as follows: prior cancer diagnosis, previous/current decline of cancer treatment, current diagnosis of major mental illness (e.g., schizophrenia) or intellectual/neurological condition, current/recent pregnancy, and residence > 70 miles from the research site. Following informed consent, participants completed questionnaires and were provided compensation ($50 gift card). See Fig. 1 for CONSORT diagram.

### Measures

#### Pain

Self-reported body and pelvic pain over the past four weeks was assessed using the International Pelvic Pain Society Pelvic Pain Assessment (PPA) – Patient version (The International Pelvic Pain Society, 2008). The 16-item questionnaire assesses pain in four areas: sexual pain (3 items), pelvic pain (4 items), overall bodily pain (4 items), menstrual pain (for the premenopausal, 4 items). Items are rated on an 11-point Likert scale to describe the level of pain in each area (0 = no pain; 10 = the worst pain imaginable). Pain scores were calculated by averaging pain ratings in each area, where higher scores indicate more pain. Internal consistency (Chronbach’s alpha) for the current study was 0.97 for the total score and 0.77–0.97 for the domain scores. As most participants indicated they were post-menopausal or not having periods at this time (93%), menstrual pain items were excluded from analyses in this study.

#### Profile of Mood States (POMS)

Anxiety was assessed using the tension-anxiety subscale of the POMS (Shacham, 1983). The eight-item subscale assesses current feelings of anxiety and tension. Items are rated on a five-point Likert scale to describe the degree to which the participant has been feeling each of the adjectives in the past week (0 = not at all; 4 = extremely). Total scores were calculated by summing all items, where higher scores indicate higher levels of anxiety. Internal consistency (Chronbach’s alpha) for the current study was 0.83.

#### Sexual avoidance

Avoidance of partner advances for sexual intercourse or activity was assessed using an investigator-created single item, “Have you avoided or declined sexual advances from your partner at all” over the past month. The item was rated on a 10-point Likert scale (0 = I have not avoided sexual activity; 10 = I have avoided sexual activity 4 or more times per day).

#### Sexual distress

Affective responses to sexual function over the past four weeks was assessed using the Female Sexual Distress Scale-Revised (DeRogatis, Leonard, Clayton, Lewis-D’Agostino, Wunderlich, & Fu, 2008; Derogatis, Leonard R., Rosen, Leiblum, Burnett, & Heiman, 2002). Thirteen items are rated on a five-point Likert scale (0 = never; 4 = always). Total scores were calculated by summing all items; higher scores indicate higher distress related to sexuality. Internal consistency (Chronbach’s alpha) for the current study was 0.95. A clinical cutoff score of ≥ 11 has shown sensitivity, specificity, and positive predictive value for identifying sexual distress among women with hypoactive sexual desire disorder (DeRogatis, Leonard, Clayton, Lewis-D’Agostino, Wunderlich, & Fu, 20082008).

### Analytic procedure

Descriptive statistics were calculated for gynecologic cancer survivors, breast cancer survivors, and the entire group; we also tested between group differences (breast vs. gynecologic cancers). Correlations between sociodemographic, disease/treatment-related variables, and the sexual distress variable were also examined to identify possible covariates. Intercorrelations were also conducted to investigate the relationship between FA model variables (i.e., pain, anxiety, and sexual avoidance). As sexual distress was not significantly correlated with ratings of pelvic or overall body pain, these pain ratings were not included in further analyses. For the main analysis, the relationship between sexual pain, anxiety, avoidance, and distress was examined using a linear regression model. Specifically, average sexual distress was included as the outcome variable and sexual pain, anxiety, and sexual avoidance were included as predictor variables. As 32% of women reported not being sexually active, this variable was added as a dichotomous covariate in the second step of the multiple linear regression analysis. All analyses were conducted using SPSS v. 28.

## Results

### Descriptive data

Participants in this study reported an average FSDS score of 17.07, which reflects more distress than in prior studies of healthy controls(Peters et al., 2007) and long-term breast cancer survivors (Raggio et al., 2014). However, the scores of this study were lower (i.e., lower levels of distress) than the average baseline scores from another psychosexual intervention study for gynecologic cancer survivors (23.19–25.44; (Brotto et al., 2012), perhaps due to differences in eligibility criteria (i.e., required to be in a current romantic relationship and reporting low sexual desire/arousal versus the less stringent eligibility criteria in the current study). Further, 50.5% (*n* = 49) of participants scored at or above the clinical cut-off of 11, indicating significant levels of sexual distress. The average PPA sexual pain rating in this sample was 1.54. Specifically, 29% reported some to the worst imaginable burning vaginal pain with sexual activity, 34% reported some to the worst imaginable deep pain with intercourse, and 18% reported some to the worst imaginable pelvic pain lasting hours or days after intercourse/sexual activity. The responses for the sexual avoidance question ranged from 0-, and average of 1.54 (note that score of 1 = sexual activity occurred once, and score of 2 = participant avoided partner advances for sexual intercourse or activity 1–2 times per month over the past three months). The average POMS Anxiety score was 9.91. No significant correlations were found between the three predictor variables. Univariate analyses were also conducted and found no significant associations between demographic characteristics and the outcome variable (e.g., sexual distress). See Table 1 for descriptive data and Table 2 for correlations.

**Table 1:** Descriptive statistics

Mean (SD)/*n* (%)			
	All participants (*n* = 97)	Gynecologic (*n* = 68)	Breast (*n* = 29)
Age	49.88 (10.75)	49.91 (10.41)	49.79 (11.74)
Race			
Caucasian/White	80 (82)	60 (88)	20 (69)
African America/Black	13 (14)	6 (8)	7 (24)
Asian	1 (1)	1 (2)	0 (0)
Other	1 (1)	1 (2)	0 (0)
Unknown	2 (2)	0 (0)	2 (7)
Highest level of education			
Associates degree/higher	63 (65)	41 (60)	22 (76)
Less than associates degree	34 (35)	27 (40)	7 (24)
Marital status			
Single/not partnered	19 (20)	12 (18)	7 (24)
Married/partnered	77 (79)	56 (82)	21 (73)
Unknown	1 (1)	0 (0)	1 (3)
Sexually inactive	31 (32)	22 (32)	9 (31)
Years since diagnosis*	2.65 (4.19)	1.58 (1.66)	5.44 (6.83)
Stage			
I	62 (64)	46 (67)	16 (55)
II/III	35 (36)	22 (33)	13 (45)
Treatment**			
Surgery	96 (99)	68 (100)	28 (97)
Radiation	33 (34)	14 (21)	19 (66)
Chemotherapy	53 (55)	33 (49)	20 (69)
Hormonal therapy	19 (20)	2 (3)	17 (59)
FSDS Total Score	17.07 (12.54)	18.20 (12.62)	14.45 (12.16)
		*t(94) = 1.35, p =.18*	
Average Sexual Pain	1.54 (2.29)	1.68 (2.60)	1.22 (1.37)
		*t(63) = 0.75, p =.45*	
POMS Anxiety	9.91 (5.97)	10.32 (5.42)	8.80 (7.29)
		*t(91) = 1.09, p =.28*	
Average Sexual Avoidance	1.54 (1.66)	1.65 (1.76)	1.24 (1.26)
		*t(81) = 0.97, p =.34*	

*p <.001

**Some participants had multiple treatments, thus totals > 100%

**Table 2:** Correlation coefficients between demographics, outcome variable, and predictor variables

	FSDS Total Score	Average Sexual Pain	POMS Anxiety	Average Sexual Avoidance
Age	− 0.04	− 0.01	− 0.19	− 0.003
Race	0.02	0.04	0.15	0.12
Highest level of education	0.01	− 0.003	− 0.11	0.11
Marital status	0.08	0.14	0.02	0.04
Sexually inactive	0.08	− 0.24	0.05	0.02
Years since diagnosis	0.04	− 0.004	0.03	0.07
Stage	0.01	0.09	− 0.12	0.01
Treatment				
Surgery	− 0.08	0.00	− 0.03	− 0.10
Radiation	− 0.18	− 0.22	− 0.22*	− 0.21
Chemotherapy	0.01	0.02	− 0.04	− 0.02
Hormonal therapy	0.05	− 0.05	0.11	− 0.13
FSDS Total Score	-	0.46**	0.39**	0.38**
Average Sexual Pain		-	0.05	− 0.35**
POMS Anxiety			-	0.21
Average Sexual Avoidance				-

*p <.05

**p <.001

### Relationship between sexual pain, avoidance, anxiety, and sexual distress

As no significant associations we found between demographic characteristics and the outcome variable (e.g., sexual distress), none were included in the multivariate analysis. Further, given the possible differences in underlying mechanisms of sexual functioning between gynecologic and breast cancer survivors, we tested for differences in all FA model components between the two groups and found no difference. Thus, the main analysis was conducted as one group and diagnosis was not included in the analysis. Sexual distress was significantly associated with all three predictor variables. These relationships remained significant when controlling for sexual activity (i.e., sexually active versus not sexually active). The models accounted for approximately 36–37% of the variance in sexual distress scores. See Table 3 for multiple linear regression model results.

**Table 3:** Multiple linear regression analysis results (*n* = 97)

Dependent: FSDS Total Score						
Variable	β	S.E.(β)	Standardized β	t	p-value	
Step 1						
Average Sexual Pain	1.97	0.64	0.35	3.09	0.003	
POMS* Anxiety	0.61	0.23	0.28	2.62	0.011	
Average Sexual Avoidance	2.41	0.99	0.28	2.43	0.019	
Adjusted *R*^*2*^ 0.366						
Step 2						
Sexually Active	-2.17	5.78	-0.04	-0.38	0.71	
Average Sexual Pain	1.91	0.66	0.34	2.89	0.006	
POMS Anxiety	0.61	0.23	0.28	2.60	0.012	
Average Sexual Avoidance	2.37	1.00	0.28	2.36	0.022	
Adjusted *R*^*2*^ 0.356						

* Profile of Mood States (POMS)

## Discussion

Pain during sexual intercourse (Bergmark et al., 1999, 2002; Bober & Varela, 2012; Jensen et al., 2004; Lindau et al., 2007; Pieterse et al., 2006) is one of the most common sexual complaints among breast and gynecologic cancer survivors. Consistent with previous studies, participants reported higher levels of sexual distress compared to healthy women, (Peterson et al., 2007) with the majority of survivors reporting sexual distress scores in the clinical range. As expected, sexual distress was associated with higher levels of acute sexual pain, anxiety, and sexual avoidance in this sample. Further, this relationship seems to be unique to occurrences of pain during sexual activity and not pelvic or general body pain.

These overall results are consistent with the FA model of chronic pain but may not fully capture the nuances of how chronic pain is associated with functioning in this patient population. Of note, our study investigated a subset of the full FA model of chronic pain that may be more likely to be assessed in typical clinical settings. Our results did not show a significant relationship between all the predictor variables (e.g., there was not a significant relationship between sexual avoidance and anxiety) as would be expected from the traditional FA model. While the FA model has been shown to be applicable to many chronic pain conditions (Vlaeyen & Linton, 2012), it may not fully account for the complexities associated with the processes that people take to resume regular tasks (Crombez et al., 2012). This may be of particular importance for this study as all participants were enrolled in a psychosexual intervention, and thus likely in the process of trying to resume or better their sexual functioning even prior to the start of the intervention. Despite this, the FA model could be used to help to identify those at risk for developing chronic problems with sexual pain. Many interventions for sexual functioning among female cancer survivors do not specifically address pain that occurs during intercourse aside from listing this as a potential side effect of treatment and recommending the use of lubricants during sexual activity (Xu et al., 2023). While some interventions have included exercises aimed at reducing pelvic floor tension (Jun et al., 2011), interventions do not address the fear of pain with sexual activity or cognitive strategies to help address this fear. While some interventions utilized cognitive behavioral therapy for general sexual functioning, desire, or body image, the authors are not aware of any that focus on pain during sexual activity. Interventions that specifically address anxiety and avoidance of sexual activity due to pain through therapies often used in other chronic pain conditions (e.g., cognitive behavioral therapy or acceptance and commitment therapy) may help with thoughts around the experience of sexual pain. Such interventions may be helpful in interrupting the chronic pain cycle and reduce the likelihood of pain during future sexual encounters.

There are some aspects of sexual pain that differentiate it from other musculoskeletal pain and should be considered, such as partner responses and the physiological mechanisms of the sexual response cycle (Thomtén & Linton, 2013). However, this study is a critical first step in highlighting the applicability of the FA model to sexual pain among female cancer survivors. Future studies should incorporate these components within a longitudinal model to assess the impact of the FA framework on the development of chronic sexual pain over time.

This study examines factors associated with sexual dysfunction from a theory-driven model among patients at risk for poor sexual functioning. However, this study has limitations that should be addressed. The sample was mostly Caucasian and endorsed high levels of education, possibly limiting the generalizability of the results; nonetheless, the distribution of ethnic minorities in our sample is comparable to the epidemiology of these diseases (Street, 2019). Further, only one participant in this study identified as non-heterosexual and thus these results may be limited by the underrepresentation of the sexual minority population. This study also used assessments that may be more representative of typical clinical interactions, rather than measures that are commonly used in research and may limit the generalizability of these findings to similar studies. For example, pain was assessed using a clinical measure not often utilized in research (PPA). While this index has not been subjected to psychometric validation, it does assess multiple aspects of pain that are especially important in this patient population (e.g., sexual pain, pelvic pain).

In addition, the purpose of this study was to investigate the relationships between some, but not all, components of the FA model of chronic pain. We did not measure pain catastrophizing specifically, rather we measured anxiety as it is highly correlated with pain catastrophizing (Sullivan et al., 1995) and is more likely to be measured in clinics part of their standard patient reported outcome assessments than pain catastrophizing. However, the findings of this model suggest that the FA model of pain catastrophizing may be applicable to this patient population. Future studies should incorporate all components of the FA model to better understand the roles that these have in sexual functioning among female cancer survivors.

Further, we assessed sexual avoidance using a single item to better align with typical interview questions in clinical interactions that focus on cancer survivorship and sexuality. Research using clinically relevant tools may be more applicable to practice settings where clinical assessments like these are utilized more often than more stringent measures. Finally, the participants in this study were enrolled in a group-based psychosexual intervention trial. It is possible that these women differ from other female cancer survivors that have not sought treatment for sexual concerns and pose the possibility of self-selection bias. For example, the participants in this study may have more impaired sexual functioning or higher levels of distress related to sexual concerns, and the findings may not generalize to survivors who are not seeking treatment.

To the authors’ knowledge, this is the first study to investigate relationships between FA components and sexual function in female cancer survivors, making it an important contribution to the field of sexuality in female cancer survivorship. Further, this study did not utilize strict eligibility criteria which may make the results more representative of clinical populations presenting to oncology or sexuality treatment settings.

In conclusion, the results of this study suggest that the FA model of chronic pain may be an applicable framework to understand the development and maintenance of chronic pain during sexual intercourse. These findings further point to the need for interventions to explicitly address the components of the FA model of chronic pain assessed in this study (e.g., anxiety, avoidance of sexual activity) and to assess the applicability of pain catastrophizing specific to sexual pain as another possible intervention target among female cancer survivors.

## Data Availability

The data that support the findings of this study are available from the corresponding author, Lora Black, upon reasonable request.

## Code Availability

n/a.
